# Redox preconditioning deep cratonic lithosphere for kimberlite genesis – evidence from the central Slave Craton

**DOI:** 10.1038/s41598-017-00049-3

**Published:** 2017-02-14

**Authors:** G. M. Yaxley, A. J. Berry, A. Rosenthal, A. B. Woodland, D. Paterson

**Affiliations:** 1 Research School of Earth Sciences, The Australian National University, Canberra, ACT 2601 Australia; 2 Bayerisches Geoinstitut, Universität Bayreuth, 95440 Bayreuth, Germany; 3 Institute of Petrology and Structural Geology, Charles University in Prague, Albertov 6, 128 43 Praha 2, Czech Republic; 4 Institut für Geowissenschaften, Goethe Universität, 60438 Frankfurt am Main, Germany; 5 Australian Synchrotron, Clayton, Victoria 3168 Australia; 6 Laboratoire Magmas et Volcans, Université Blaise Pascal, CNRS IRD-OPGC, Campus Universitaire des Cézeaux, 6 Avenue Blaise Pascal, 63178 Aubière Cedex, France

## Abstract

We present the first oxygen fugacity (*f*O_2_) profile through the cratonic lithospheric mantle under the Panda kimberlite (Ekati Diamond Mine) in the Lac de Gras kimberlite field, central Slave Craton, northern Canada. Combining this data with new and existing data from garnet peridotite xenoliths from an almost coeval kimberlite (A154-N) at the nearby Diavik Diamond Mine demonstrates that the oxygen fugacity of the Slave cratonic mantle varies by several orders of magnitude as a function of depth and over short lateral distances. The lower part of the diamond-bearing Slave lithosphere (>120–130 km deep) has been oxidized by up to 4 log units in *f*O_2_, and this is clearly linked to metasomatic enrichment. Such coupled enrichment and oxidation was likely caused by infiltrating carbonate-bearing, hydrous, silicate melts in the presence of diamond, a process proposed to be critical for “pre-conditioning” deep lithospheric mantle and rendering it suitable for later generation of kimberlites and other SiO_2_-undersaturated magmas.

## Introduction

The *f*O_2_ of the Earth’s deep interior is critically important, influencing diverse processes in the solid and volatile cycles of our planet. It controls the speciation of volatile components (CHONS) in the mantle, including diamond/graphite *versus* carbonate stability. This profoundly influences melting temperatures and types of partial melts of mantle rocks and the nature of mantle metasomatism. It affects volatile solubilities in magmas and hence outgassing of CHONS-volatiles and so was influential in the formation and nature of the atmosphere. Knowledge of the distribution of the mantle’s *f*O_2_ with depth, location and time is critical for understanding large-scale volatile cycles and fluxes between the crust, ocean and atmosphere, and deep Earth (mantle, core)^1^.

In the absence of partial melts or fluids, the *f*O_2_ of the Earth’s upper mantle is controlled internally by redox sensitive reactions involving Fe-bearing mineral components in spinel, garnet and pyroxenes in which Fe has variable oxidation states. Because of the positive molar volume changes of these reactions^2–4^ *f*O_2_ should decrease steadily with increasing pressure (depth) in the upper ≈250 km of the peridotite-dominated mantle. At pressures around 8–9 GPa (≈250–300 km deep) the depth-*f*O_2_ trend should intersect the Ni precipitation curve, and an FeNi metallic alloy is expected to form^4^. The *f*O_2_ of peridotite will then remain close to that of the Ni precipitation curve (≈IW to IW-1 log unit)^5^ down to the transition zone at ≈410 km depth^4^.

Experimental calibrations of redox controlling reactions in garnet peridotite assemblages^2, 3^, coupled with determinations of the Fe^3+^/∑Fe of garnet from peridotite xenoliths transported by kimberlite magmas to the surface from depths as great as 220 km (~7 GPa), enable determination of the *f*O_2_ of the cratonic mantle lithosphere. There is now data from several different cratons^6–13^.

We have determined the oxygen fugacities recorded by garnet peridotite xenoliths hosted by two nearly coeval and diamondiferous kimberlites from the geographically close Ekati and Diavik mine leases in the central Slave Craton, Canada. In combination with conventional thermobarometry, this allows construction of the first *f*O_2_ profile through the cratonic lithospheric mantle under the Ekati Diamond Mine and demonstrates that the *f*O_2_ of the Slave cratonic mantle can vary by several orders of magnitude as a function of depth and over short lateral distances. The implications for kimberlite genesis are then discussed. No redox data has previously been reported from xenoliths from the Panda kimberlite.

## Results and Calculations

### Sample locations and other details

Fresh garnet peridotite xenoliths recovered from the Panda kimberlite on the Ekati Diamond Mine lease and from the A154-North (A154-N) pipe on the nearby Diavik lease were investigated. The Panda samples consist of 30 fragments of peridotite approximately 1 cm across, derived from fragmentation of larger xenoliths during processing at the mine. In all cases, they contain relict, fresh primary mineral phases. Major and minor element compositions for all mineral phases were presented by Menzies *et al.* ^14^. The Diavik suite includes 10 new and very fresh garnet lherzolite xenoliths recovered from the A154-N kimberlite pipe at the Diavik Diamond Mine in Lac de Gras, Northwest Territories, Canada. The data from these new samples is presented in Supplementary Tables 1–5 and is supplemented by earlier data from other garnet peridotite xenoliths from the same pipe^8^. The two host kimberlites were emplaced at 53.3 ± 0.6 Ma (Panda)^15^ and 56.0 ± 0.7 Ma (A154-N)^16^, and are located about 30 km apart near the edge of Lac de Gras in the central Slave Craton (see Fig. 1 of Creighton *et al.* ^8^ for a location map).

**Figure 1:**
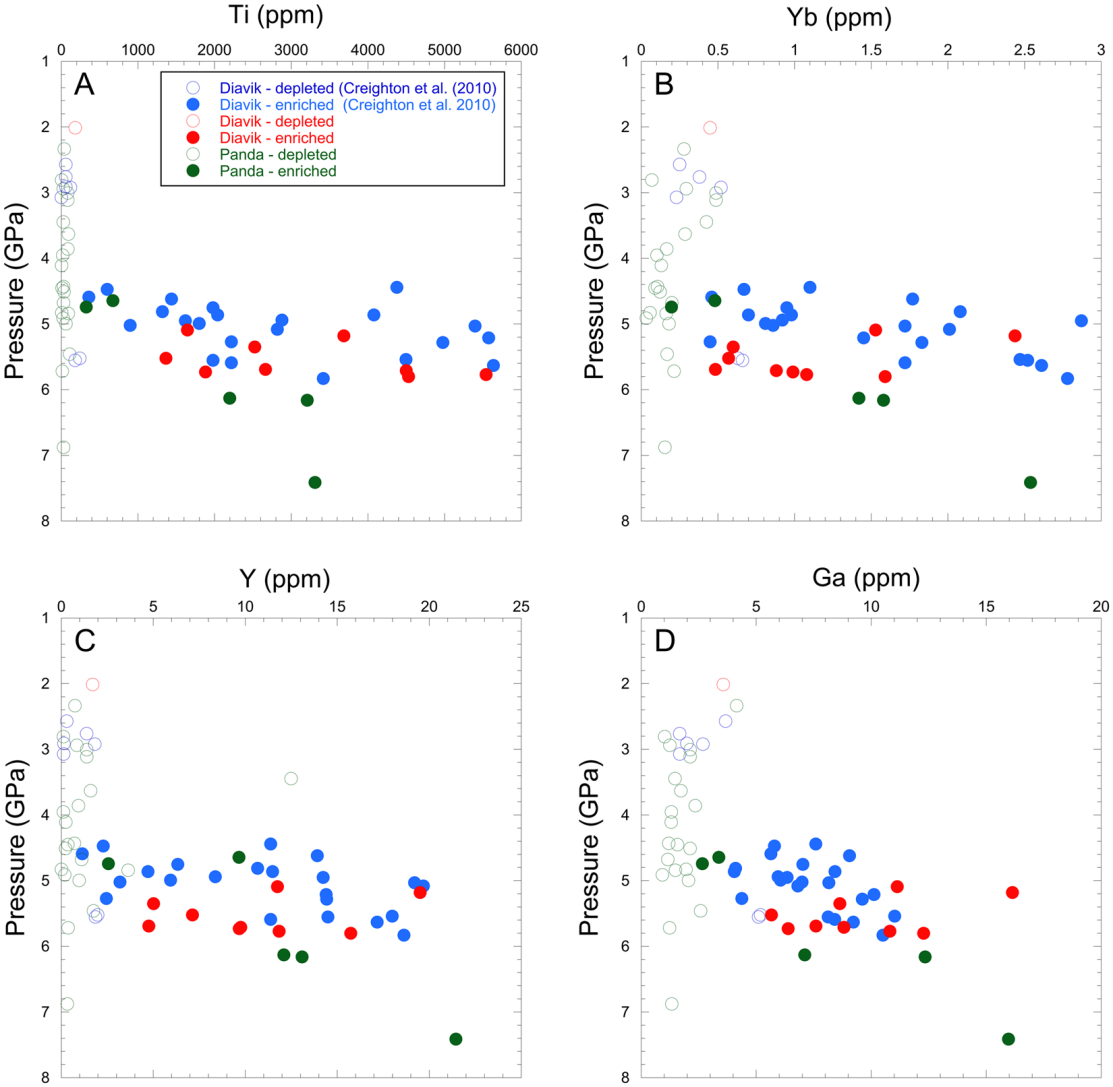
Garnet (**A**) Ti, (**B**) Yb, (**C**) Y and (**D**) Ga *vs*. pressure (GPa) for the Panda and A154-N samples. Data for some A154-N samples are from Creighton *et al.* ^8^. Depleted samples contain garnet with <200 ppm Ti, following Griffin and Ryan^21^.

### Major, minor and trace element compositions of mineral phases

Electron microprobe analyses of the major and minor element abundances and laser-ablation inductively couple plasma mass spectroscopy (LA-ICPMS) analyses of trace element abundances in constituent phases in the new A154-N garnet lherzolite xenoliths are presented in Supplementary Tables 1–4. The data presented are averages of multiple analyses of each phase in each sample. In Supplementary Table 4 Fe^3+^/∑Fe data for garnets from the A154-N (Diavik) suite, determined by Mössbauer spectroscopy, are presented and the wt% oxide and cation analyses have incorporated this data. In Supplementary Table 5 Fe^3+^/∑Fe data for garnets from the Panda (Ekati) suite, determined by X-ray Absorption Near-Edge Structure spectroscopy (XANES), are presented. Major and minor element chemistry for garnets from Menzies *et al.* ^14^ are also re-presented, but with Fe_2_O_3_ and Fe^3+^ included in the wt% oxide and cation analyses respectively, calculated using the XANES measurements. All XANES Fe^3+^/∑Fe measurements are presented. New LA-ICPMS garnet trace element data are also presented.

Major element chemistry of mineral phases in the Panda samples and in some of the A154-N samples has been comprehensively described previously^8, 14^. The mineral chemistry of the new A154-N samples is typical of other fertile garnet peridotite xenoliths from various locations around the world. Olivine Mg# (where Mg# = 100*Mg/[Mg+Fe]) varies from 90.0 to 92.9. All new A154-N samples are lherzolites and clinopyroxene is Cr-diopside-rich with Mg# ranging from 90.5 to 95.2, Na_2_O from 0.94 to 1.97 wt% and TiO_2_ up to 0.23 wt%. Orthopyroxene has Mg# from 91.2 to 93.6, CaO from 0.22 to 1.09 wt% and Al_2_O_3_ from 0.41 to 0.70 wt%. Garnets contain from 1.77 to 9.90 wt% Cr_2_O_3_, 4.32 to 6.95 wt% CaO and are classified as lherzolitic G9 types^17^. CaO and Cr_2_O_3_ are very well correlated. Fe^3+^/∑Fe was determined using Mössbauer spectroscopy and ranges from 0.022 to 0.105, similar to other suites of garnet peridotites^7, 10, 18^. Mineral grains from all samples are homogenous within analytical uncertainty on an intra- and intergrain basis.

Trace element abundances in mineral phases present in the samples from the Panda kimberlite and the new samples from the A154-N kimberlite were determined using LA-ICPMS (see Methods) and are presented in Supplementary Tables 2, 4 and 5. Data for the remaining A154-N samples of Creighton *et al.* has not been presented^8^.

### Thermobarometry of the garnet peridotites

Based on conventional thermobarometry^19, 20^, the Panda samples equilibrated at pressures from 2.3 ± 0.3 to 6.9 ± 0.3 GPa and temperatures from 717 ± 20 to 1271 ± 20 °C. The A154-N samples equilibrated at pressures from 2.0 ± 0.3 to 5.8 ± 0.3 GPa and temperatures from 457 ± 20 to 1269 ± 20 °C (Supplementary Table 6). The coeval and geographically close nature of the Panda and A154-N kimberlites and the sampling of material over a depth interval that includes most of the vertical lithospheric section, afford an excellent opportunity to assess lateral and vertical heterogeneity in cratonic lithospheric mantle down to nearly 7 GPa (≈200 km depth).

### Trace element mineral chemistry of the garnet peridotites

Abundances of incompatible trace elements in garnet (and clinopyroxene) are presented in Supplementary Tables 2, 4 and 5. Abundances of trace elements such as Y, Yb, Ga and Ti in garnet have long been recognized as indicators of the relative depleted versus enriched nature of garnet peridotite xenoliths^21^. When abundances of these elements in garnet are plotted against pressure (P), it is clear that the Slave lithosphere under the A154-N pipe is highly depleted at depths shallower than ≈135 km (4.5 GPa) (Fig. 1). At greater depths, the garnets contain a much larger range and mostly higher abundances of these elements, indicating that the material present is variably and often strongly enriched. Under the Panda pipe at the nearby Ekati Diamond Mine, the lithospheric architecture is broadly similar to that under the nearby A154-N pipe, although the proportion of samples containing enriched garnet is lower. Also, at Ekati more depleted material was sampled from P > 4.5 GPa than was the case at Diavik. As for A154-N, Panda garnets derived from P ≤ 4.5 GPa are exclusively depleted in these trace elements relative to most higher-pressure garnets (Fig. 1).

**Figure 2:**
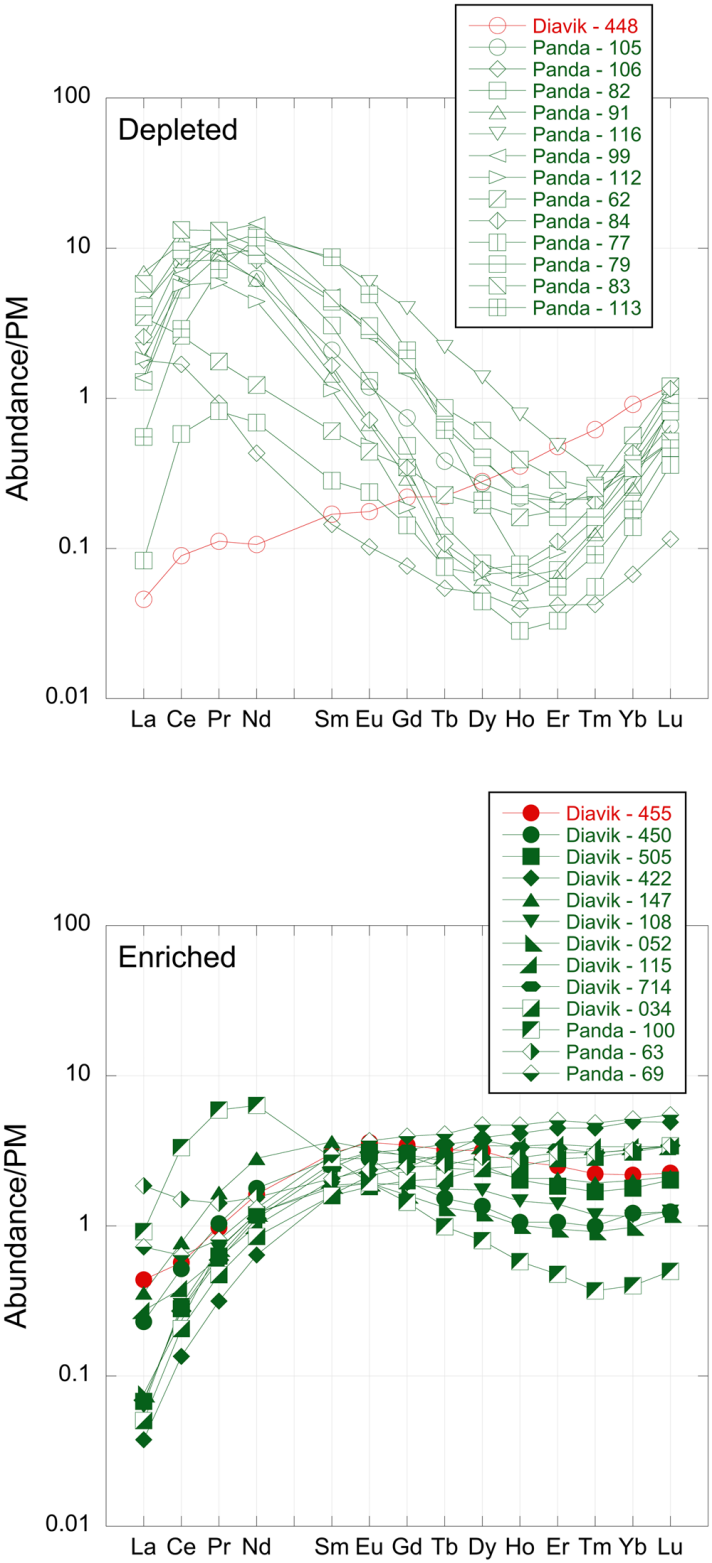
Primitive Mantle normalized^60^ garnet REE patterns from depleted and enriched garnets from the Panda and new A154-N samples. Depleted garnets contain <200 ppm Ti. Nearly all depleted garnets have strongly sinusoidal REE patterns, whereas the majority of enriched garnets have normal patterns. Sample numbers are indicated in the key. In the case of the Diavik samples, the samples numbers quoted in the key correspond to the last three digits of the full sample numbers listed in Supplementary Table 4.

Cratonic lithospheric garnet normalized Rare Earth Element patterns are distinctive – “sinusoidal” patterns are usually found in garnets in more depleted lithologies (harzburgites) but “normal” patterns are usually in more fertile lherzolitic lithologies^22^. This is also observed in the current samples in that the shallower, depleted samples mostly exhibit “sinusoidal” patterns and the deeper, enriched samples have “normal” patterns (Fig. 2). The Slave peridotitic garnet compositions therefore indicate strikingly that the upper part of the lithosphere (P ≤ 4.5 GPa) is depleted, but at P > 4.5 GPa, both depleted and variably enriched material is present. Thus, the central Slave cratonic lithosphere is characterized by two layers, the upper depleted and the lower containing both enriched and depleted components, with a remarkably sharp contact between the two at a depth of ≈140 km. Broadly similar inferences were made by Griffin *et al.* ^23^ based on garnet and Cr-spinel heavy mineral concentrates from Cretaceous-Tertiary central Slave Craton kimberlites.

### Oxygen fugacity determinations

Oxygen fugacity conditions experienced by the xenoliths prior to entrainment in the host kimberites were calculated using the garnet peridotite oxybarometer of Stagno *et al.* ^3^. Relative to the fayalite-magnetite-quartz (FMQ) redox buffer calculated for the pressure-temperature (PT) conditions of equilibration of each mantle xenolith, we obtained Δlog*f*O_2_[FMQ] from −0.47 to −3.89 log units (±0.6 log units) for the Panda suite and 0.18 to −3.36 log units for the new A154-N suite (Supplementary Table 6). Combining the new A154-N data with those from Creighton *et al.* ^8^, recalculated using the Stagno *et al.* ^3^ calibration, defines a range of Δlog*f*O_2_[FMQ] from +0.95 to −4.11 log units over a PT range of 2.01 to 6.85 GPa and 457 to 1346 °C for the full Diavik suite.

## Discussion

**Figure 3:**
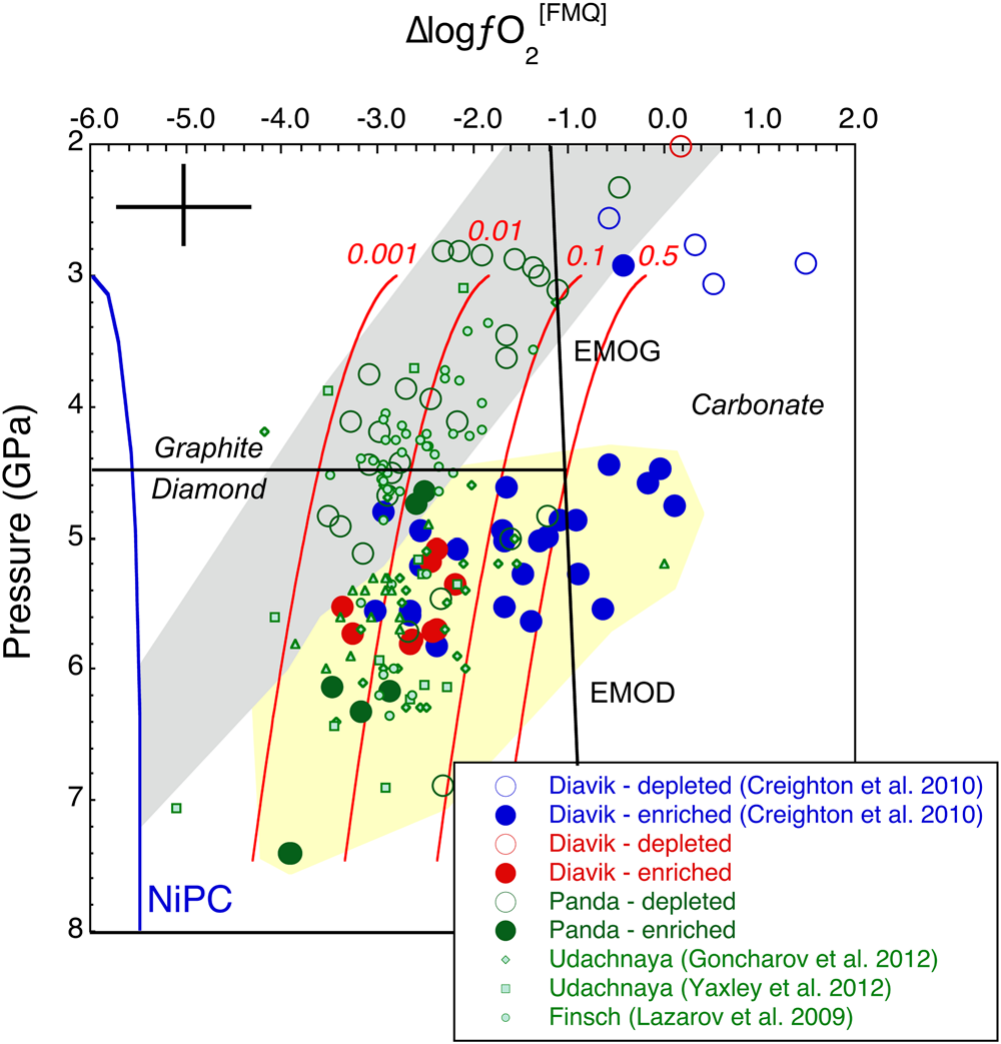
Δlog*f*O_2_[FMQ]^3^ *vs*. pressure in GPa for the current and published, depleted or enriched garnet peridotite xenolith data from the Slave, Kaapvaal and Siberian Cratons^6–8, 10, 61^. Grey field encompasses almost entirely depleted Slave Craton samples and the yellow field mostly enriched Slave Craton samples. NiPC is the Ni precipitation curve^5^. The pressure of the graphite-diamond transition was determined from Kennedy and Kennedy^62^ assuming a 35 mWm^−2^ cratonic geotherm^63^. EMOG and EMOD refer to limiting reactions for carbonate/diamond stability in harzburgitic assemblages (enstatite + magnesite = forsterite + graphite/diamond + O_2_)^3, 64^, calculated along an assumed 35 mWm^−2^ cratonic geotherm. The red curves are contours of the relationship between oxygen fugacity and pressure for equal mole fractions of CO_2_ in silicate melts (indicated by the red numbers next to each curve) calculated following Stagno *et al.* ^25^. The black cross in the upper left corner indicates the estimated uncertainties in the *f*O_2_ and pressure calculations, based on Stagno *et al.* ^25^ and Nimis and Grütter^20^.

In Fig. 3, the variation in *f*O_2_ as a function of depth for the A154-N and Panda samples is plotted, revealing several features;

1. Δlog*f*O_2_[FMQ] varies by up to ≈4 log units at almost any depth; Lateral heterogeneity in oxygen fugacity of 3 log units has also been observed in the northern part of the Slave Craton using eclogite and pyroxenite xenoliths^24^.
2. The shallow, depleted Panda samples and some depleted A154-N samples define a broad trend of decreasing *f*O_2_ with increasing pressure down to 5.0 GPa.
3. At P > 4.5 GPa, there is a trend to more oxidized values than expected by deeper extrapolation of the lower pressure *f*O_2_-P trend (grey field on Fig. 3). Samples defining this oxidized zone (yellow field on Fig. 3) include nearly all enriched A154-N samples, all enriched Panda samples and 5 of the depleted Panda samples. These 5 depleted Panda samples were classified as depleted on the basis of their low garnet Ti contents (<200 ppm), but they are relatively enriched in Yb, Y and Ga (Fig. 1). Hence, this deep, oxidized domain corresponds quite precisely to the trace element enriched layer in the lower part of the lithosphere and to the diamond stability field defined in P-T-*f*O_2_ space, although 8 of the A154-N samples of Creighton *et al.* ^8^ lie in the carbonate stability field (Fig. 3).

Thus, although the central Slave cratonic lithosphere at P < 4.5 GPa decreases in *f*O_2_ with increasing pressure as predicted by thermodynamic considerations, at P > 4.5 GPa, there are no samples with *f*O_2_ values on the extrapolation of this trend to higher pressures. Instead almost all samples from these depths are at least 1–4 log units higher in *f*O_2_. Given that the majority of these deep and oxidized samples are enriched relative to the samples from shallower depths (P < 4.5 GPa), we argue that they have been metasomatised and that the metasomatism was strongly oxidising. This effectively requires addition of oxygen to the deep cratonic lithosphere. Likely candidates for the responsible metasomatic agents are carbonate-bearing, silicate melts^3, 25^, possibly derived from the deeper, reduced asthenosphere^26^ (at >230 km depth, i.e. P > 7 GPa), or saline, hydrous fluids derived from a postulated subducted slab beneath the base of the Slave cratonic lithosphere^27^. Such melts may contribute to the commonly observed elevated modes of orthopyroxene in some garnet peridotite xenolith suites^28^, in a manner similar to that proposed by Kelemen^29^.

At these pressures the mantle may be still under-saturated in metallic FeNi^4^. Stagno *et al.* ^3, 25^ showed that silicate melts with dilute carbonate contents could be stable at the relatively reduced *f*O_2_ values of the mantle at these depths because of the lower carbonate activity relative to that of low-SiO_2_ carbonatite melts (Fig. 3). If these carbonated, silicate melts segregate and percolate upwards through parts of the deep cratonic lithosphere they would encounter progressively cooler conditions along the cratonic geotherm, at some point freezing into the lithospheric mantle as the appropriate peridotite + volatile solidus is locally crossed^30^. The oxidized carbon species exsolved from the melt during crystallisation would reduce to C or CH_4_ (CO_2_ = C + O_2_; CO_2_ + 2H_2_O = CH_4_ + 2O_2_). Very minor amounts of such C and H might be incorporated into nominally volatile-free, peridotitic silicate minerals (such as olivine, ortho- and clinopyroxene, garnet)^31, 32^, or if in excess, may form CH_4_ and/or diamond when in contact with reduced deep peridotite. Some of the Fe^2+^ in the melt and wall rock would oxidize to Fe^3+^ (2FeO + 1/2 O_2_ = Fe_2_O_3_). This and other components would be incorporated into garnet and pyroxenes, leading to the observed increase in lithospheric *f*O_2_ associated with metasomatism. The increased activity of H_2_O may cause a substantial drop in the solidus temperature of peridotite and consequent partial melting, if the quantity of newly formed H_2_O exceeds the storage capacity of nominally anhydrous minerals in peridotite at given pressure, temperature and *f*O_2_ (refs 30 and 33), a process known as hydrous redox melting^34, 35^.

An alternative scenario for metasomatism of the deep Slave cratonic lithosphere has been proposed by Weiss *et al.* ^27^, whereby hydrous, carbon-bearing and saline fluids in a subducted slab underlying the cratonic lithosphere percolate into the overlying lithosphere, evolving to silicic and carbonatitic melts which are preserved as inclusions in syngenetic fibrous diamonds. Similarly, Miller *et al.* ^36^ proposed a model of fibrous and non-fibrous diamond formation from reduction of carbonatitic fluids under Ekati, accompanied by enrichment of the mantle in Ca and REE. Reduction of carbonate components in such fluids to diamond could conceivably be accompanied by oxidation of Fe^2+^ in residual fluids and silicate phases which might crystallise from them.

The observations and processes described above have important general implications for the petrogenesis of kimberlites, some of which (including those discussed here) are primary hosts of economic diamond deposits. High pressure experimental investigations show that group I kimberlite magmas likely formed from low degree melting of carbonate + H_2_O-bearing garnet harzburgite at pressures near the base of typical cratonic lithosphere^37–40^, whereas group II kimberlitic magmas (orangeites) may have formed from phlogopite-bearing peridotite^41^. The pressure-temperature conditions for the origins of group I kimberlites were proposed to be those at which the primary melt is saturated in magnesite-bearing garnet peridotite, but will vary with the details of volatile contents and species^37^. Ca-bearing magnesite is most likely a necessary phase in the peridotitic melting assemblage to form primary, carbonate-rich melts which can evolve to kimberlite^37^.

This introduces a problem, because at depths corresponding to the likely source regions of kimberlites (near the base of cratonic lithosphere at ≈5–7 GPa [160–230 km], or deeper in the asthenosphere) ambient peridotite mantle oxygen fugacity is too low for crystalline carbonate stability. Predictions from thermodynamic arguments and trends from direct measurements of depleted garnet peridotite xenoliths, as presented here, show that the mantle’s *f*O_2_ at these depths (≈160–230 km) is approaching the Ni precipitation curve at around the iron-wüstite (IW) buffer^4, 7^. Hence, the pre-metasomatic, deep cratonic lithosphere may have been too reduced for carbonate to be stable as a crystalline solid solution (i.e. Ca-magnesite). Even deep lithosphere oxidized by metasomatism, as represented by the xenoliths discussed here, is mostly too reduced for crystalline carbonate stability. Rather such pressure-temperature-*f*O_2_ conditions favour the stability of diamonds as the solid carbon phase in equilibrium with a metasomatic, carbonate-poor, but water-bearing silicate melt^3^.

Tappe *et al.* ^26^ have proposed a model for genesis of the Lac de Gras kimberlites, based in part on radiogenic isotope systematics. Lac de Gras kimberlites exhibit restricted ranges in Sr and Nd isotopic compositions, close to bulk earth and CHUR values, but extreme heterogeneity in Hf isotope compositions (Δε_Hf_ varies from +5 to −5). In addition, the Lac de Gras kimberlites are known to have transported ultra-deep diamonds^42^ to the surface. The decoupling of different radiogenic isotope systems and evidence of ultra-deep components in the kimberlites has been explained by a model in which ancient, deeply recycled crustal lithologies (both MORB^43^ and OIB^26^) in the Mantle Transition Zone (MTZ) underwent partial melting. These melts refertilised refractory peridotite wall-rock in the MTZ or deep upper mantle. Vigorous mantle convection transported the refertilised domains upwards, in some cases allowing transport of ultra-deep diamonds (and their inclusions) from the deep upper mantle or MTZ, to shallower levels. Some of the refertilised domains may have reached depths at which the peridotite mantle was no longer metal saturated (≤300 km)^4^ and may have undergone redox partial melting to produce CO_2_-bearing, silicate magmas^25^. These magmas were then transported into the deep cratonic lithosphere, metasomatically enriching and oxidizing it over time.

Progressive, long term oxidation of conduits or zones in the deep cratonic lithosphere by infiltrating carbonate-bearing silicate melts, in the manner described above, or by the incorporation of subducted carbonate-bearing crustal material, and associated complex redox-melting and freezing reactions^27, 44, 45^, could lead to pressure-temperature-*f*O_2_ conditions under which crystalline carbonate phases (Ca-magnesite) become stable in deep, refractory cratonic peridotite relative to diamond or CH_4_-fluids. These may ultimately constitute *f*O_2_ heterogeneities, which could be oxidized and chemically enriched, localized sources of carbonatites, kimberlites and other highly silica-undersaturated magmas^46, 47^. This metasomatism may also add accessory phlogopite or K-richterite to the deep lithosphere^30, 48^, phases implicated in the petrogenesis of some alkali-rich magmas^41, 46, 49, 50^. Therefore, carbonatites and kimberlites may form at these depths only after long-term redox pre-conditioning of source regions near the base of the cratonic lithosphere (≈5–7 GPa) by metasomatism, where the pre-conditioning involved trace element enrichment and oxidation to levels of carbonate, rather than diamond stability.

## Methods

### Sample preparation

The Panda samples were mounted in 1-inch diameter round epoxy buttons, sectioned and polished to expose mineral phases. The new A154-N samples were presented as polished thin-sections (thickness 60 µm).

### Conventional electronprobe microanalysis

The major and minor element compositions of mineral phases from the new A154-N garnet peridotite samples were determined by wave-length dispersive electronprobe microanalysis (EPMA) using the 4–spectrometer Cameca SX100 instrument at the Australian National University (ANU). Calibration was performed using a range of well-characterised natural minerals standards. Column conditions were 15 kV and 20 nA, with the beam focused to 1 µm diameter. Peak counting times varied from 10–60 seconds, depending on element abundance, and background counting times varied from 5–30 seconds.

### Laser ablation-ICPMS

Trace element abundances in garnet and clinopyroxene in the xenoliths were measured by laser ablation ICP-MS, using the instrument at RSES, ANU. This consists of an Excimer 193 nm laser coupled to a purpose built sample cell and an Agilent 7500 ICP-MS^51, 52^.

Analytical conditions used were typically as follows. The laser spot size was varied from 135 to 225 µm depending on the nature of the phase being analysed. The laser pulsed at 5 Hz. For each analysis a gas blank (laser off) was collected for 25 seconds, the laser triggered, and the signal collected for a further 35 seconds. The ablation atmosphere was He and the ablated material was delivered to the Ar plasma in an Ar/H_2_ mixture. The ionised material was analysed in peak hopping mode by the quadrupole mass spectrometer.

The analytical protocol typically consisted of analyses of the calibrating standard (NIST612 glass) bracketing 6–10 unknowns. BCR2g glass or garnet MU53388^53^ were also analysed as unknowns to provide a check on data quality. In almost all cases, measured trace element abundances in BCR2g and MU53388 were within 5–10% of accepted values.

^43^Ca was used as the internal reference element, based on CaO determined by EPMA. The values for NIST612 of Pearce *et al.* ^54^ were used in the data reduction. A linear drift correction was applied to the background corrected signal for each analysed mass, based on interpolation between bracketing analyses of calibration standards^51^.

### Mössbauer spectroscopy

Garnet Fe^3+^/∑Fe from the new A154-N samples was measured using Mössbauer spectroscopy applied to ≈20 mg samples of clean, inclusion and alteration-free garnet fragments, separated by hand-picking under a binocular microscope from crushed portions of all xenoliths except 60505115. Garnet grains from sample 60505115 exhibited cloudy interiors as a result of the presence of abundant inclusions, and were therefore not considered suitable for bulk analysis using Mössbauer spectroscopy. Separated material from the other samples was washed for 5 minutes in HF acid to remove any surface alteration. The acid-washed grain fragments were then inspected with a binocular microscope to ensure that they were free of inclusions and alteration material. Analysis and data reduction were performed at the University of Frankfurt following well-established procedures of Woodland and Koch^10^ and Woodland and Ross^55^. Garnet grains separated from these samples have been used as calibration standards for the XANES method for determining Fe^3+^ in mantle garnet^7, 18, 56, 57^.

### X-ray Absorption Near-Edge Structure (XANES) spectroscopy

Garnet Fe^3+^/∑Fe was determined on the Panda xenoliths using the Fe K-edge XANES method of Berry *et al.* ^56^. Spectra were collected at the X-ray Fluorescence Microscopy beamline at the Australian Synchrotron^58^. The method was calibrated using homogenous garnet standards from the new A154-N samples, for which Fe^3+^/∑Fe was previously determined by Mössbauer spectroscopy and the suite of Kaapvaal samples of Woodland and Koch^10^, for which garnet Fe^3+^/∑Fe was also determined by Mössbauer spectroscopy. Up to three individual garnet grains per sample were measured.

**Figure 4:**
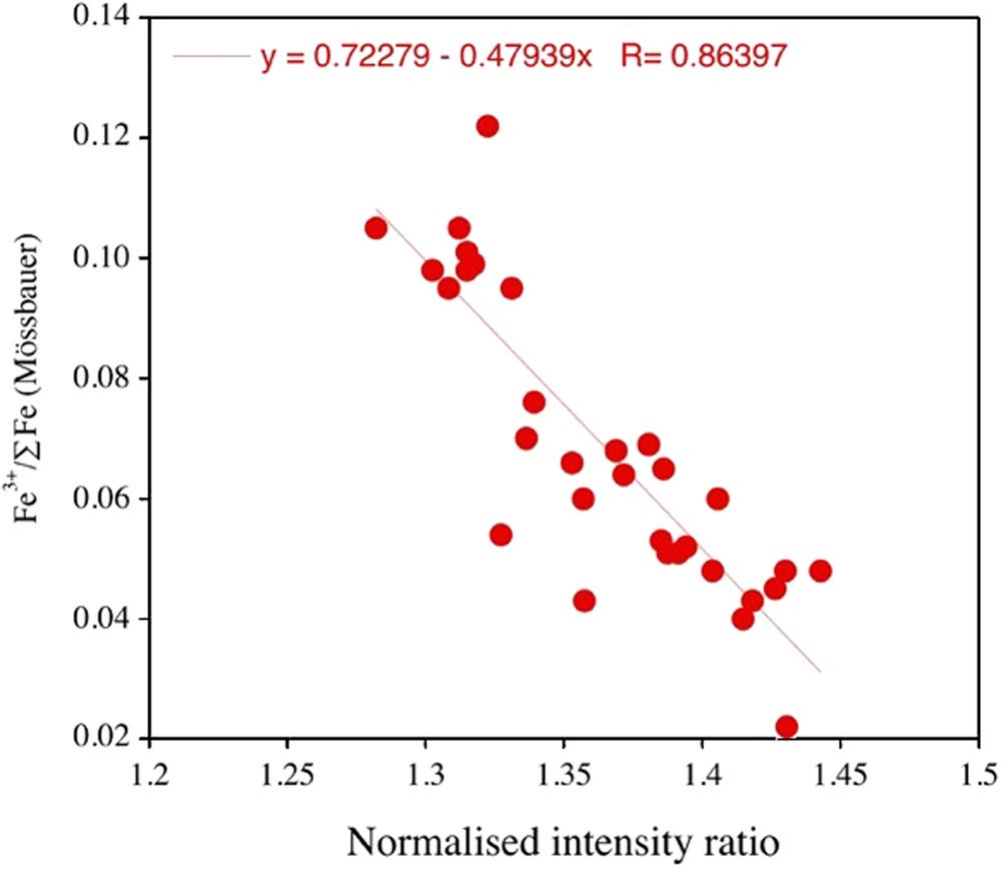
Calibration curve for the XANES measurements, relating Fe^3+^/∑Fe as determined by Mössbauer spectroscopy on standard garnets from garnet peridotite xenoliths with the normalized intensity ratio of post-edge features in the Fe XANES spectrum at 7138.4 and 7161.7 eV.

Following Berry *et al.* ^56^ the calibration curve obtained at the beamline related the normalized intensity ratios of the post-edge features in the XANES spectrum at 7138.4 and 7161.7 eV to the Fe^3+^/∑Fe of the standard garnets as determined by Mössbauer spectroscopy (Fig. 4). The precision of the XANES Fe^3+^/∑Fe measurements is estimated at ±0.012, comparable with that of the Mössbauer measurements^10^.

### Thermobarometry

Equilibration pressures and temperatures were calculated for the new A154-N and Panda xenoliths using conventional thermometers and barometers. The results are presented in Supplementary Table 6. For the A154-N samples, the approach of Nimis and Grütter^20^ was used as this has been shown to be a suitable thermometer and barometer combination for garnet lherzolite assemblages^20^. However, because many of the Panda samples do not contain modal clinopyroxene, the garnet-olivine thermometer of O’Neill and Wood^59^ combined with the garnet-orthopyroxene barometer of Brey and Köhler^19^ was preferred. Fe^3+^ in garnet determined by XANES spectroscopy was incorporated into the calculations.

### Calculation of oxygen fugacity

The presence in the new samples of fresh garnet, olivine and orthopyroxene was used as a basis for selection for garnet Fe^3+^ analysis, to enable calculation of the samples’ *f*O_2_^3^. This information (Supplementary Tables 4 and 5), combined with the results of thermobarometry and the major element compositions of co-existing olivine and orthopyroxene measured by electronprobe microanalysis^8, 14^, enabled calculation of the *f*O_2_ conditions^3^ which the xenoliths experienced prior to entrainment in the host kimberlite (Supplementary Table 6).

All *f*O_2_ calculations presented in this paper were calculated (or re-calculated) using the experimental calibration for garnet peridotite assemblages of Stagno *et al.* ^3^ and garnet Fe^3+^/∑Fe determinations by Mössbauer spectroscopy (the new samples from A154-N, following the method of Woodland and Koch^10^), or Fe K-edge XANES (the Panda samples, following the method of Berry *et al.* ^56^). This data was coupled with new mineral compositional data for the new A154-N samples, previously reported data for the Panda^14^ suite and the earlier A154-N suite^8^ and with the thermobarometric data as reported above. Uncertainties in the *f*O_2_ calculations are estimated at ±0.6 log units.

## Electronic supplementary material

Supplementary Table 1

Supplementary Table 2

Supplementary Table 3

Supplementary Table 4

Supplementary Table 5

Supplementary Table 6
